# Large-scale test data set for location problems

**DOI:** 10.1016/j.dib.2018.01.008

**Published:** 2018-01-10

**Authors:** Matej Cebecauer, Ľuboš Buzna

**Affiliations:** a Department of Transport Science, KTH Royal Institute of Technology, Teknikringen 10, SE-100 44 Stockholm, Sweden; b Department of Mathematical Methods and Operations Research, University of Žilina, Univerzitná 8215/1, SK-010 26 Žilina, Slovakia; c ERA chair for Intelligent Transport Systems, University of Žilina, Univerzitná 8215/1, SK-010 26 Žilina, Slovakia

## Abstract

Designers of location algorithms share test data sets (benchmarks) to be able to compare performance of newly developed algorithms. In previous decades, the availability of locational data was limited. Big data has revolutionised the amount and detail of information available about human activities and the environment. It is expected that integration of big data into location analysis will increase the resolution and precision of input data. Consequently, the size of solved problems will significantly increase the demand on the development of algorithms that will be able to solve such problems. Accessibility of realistic large scale test data sets, with the number of demands points above 100,000, is very limited. The presented data set covers entire area of Slovakia and consists of the graph of the road network and almost 700,000 connected demand points. The population of 5.5 million inhabitants is allocated to the locations of demand points considering the residential population grid to estimate the size of the demand. The resolution of demand point locations is 100 m. With this article the test data is made publicly available to enable other researches to investigate their algorithms. The second area of its utilisation is the design of methods to eliminate aggregation errors that are usually present when considering location problems of such size. The data set is related to two research articles: “A Versatile Adaptive Aggregation Framework for Spatially Large Discrete Location-Allocation Problem” (Cebecauer and Buzna, 2017) [1] and “Effects of demand estimates on the evaluation and optimality of service centre locations” (Cebecauer et al., 2016) [2].

**Specifications Table**

**Table:** 

Subject area	Applied mathematics, Operations research, Discrete optimization
More specific subject area	Location analysis, Geographic information systems
Type of data	graph of the road network, weighted demand points derived from GIS data and residential population grid
How data was acquired	Data set was created by combing publicly available data sets such as OpenStreetMap and residential population grid.
Data format	csv text files, shapefiles
Data source location	Slovakia (Longitude 17.001–22.110, Latitude 47.732–49.586)
Data accessibility	The data are available with this article. Moreover, data is published on the professional web page of one of the co-authors: http://frdsa.uniza.sk/~buzna/page5/page5.html

**Value of the Data**

• Data set can be used as a benchmark to design and experiment with new location algorithms intended to solve large-scale locational and spatial problems.
• Data set is applicable in the design and studies of new aggregation methods to minimise the impact of aggregation errors on the outcome of optimisation.
• Data set can be used to derive large number of medium and small size benchmarks by selecting specific geographic areas.
• Data set enables visualisation of results of optimisation algorithms in GIS.

## 1 Data

**Fig. 1:**
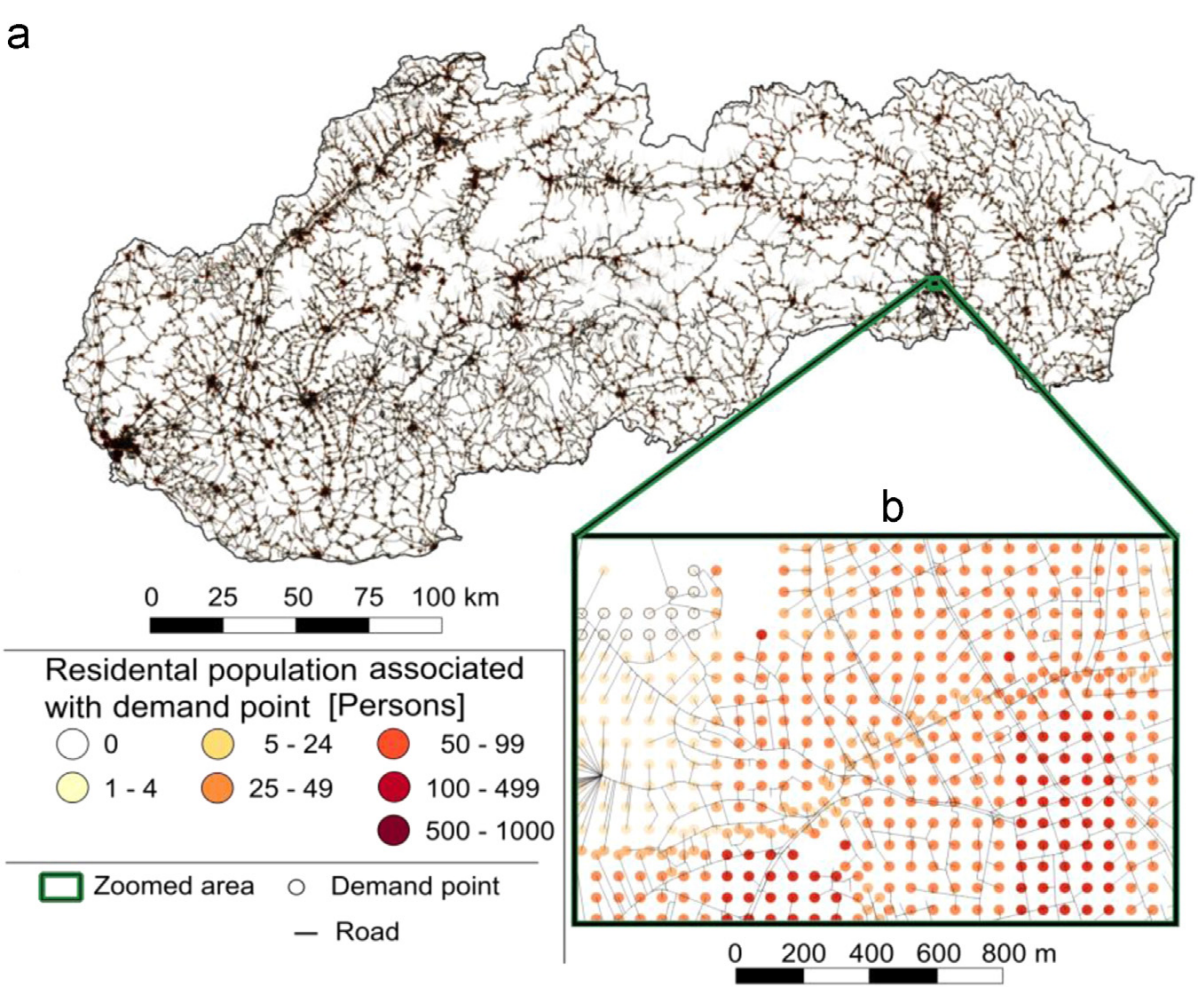
Visualization of the benchmark Slovakia. (a) Entire area of Slovakia covered by the benchmark. (b) Zoom illustrating the resolution of the model.

Central component of the benchmark Slovakia is the graph consisting of 1,956,067 georeferenced nodes, further defining 2,080,694 edges, representing the road sections covering the entire area of Slovakia. Some of these nodes ( 663,203) identify the potential population demand distribution derived from the residential population density. In the literature it is common to refer to these points as to demand points (DPs). A potential demand is located in the populated area approximately each 100 m and connected to the road network (see Fig. 1 for illustration).

With this article we deliver four different benchmarks (see Table 1), the main benchmark Slovakia and three benchmarks, Žilina, Košice and Partizánske. Benchmarks Žilina, Košice and Partizánske has been derived from the benchmark Slovakia by restricting the scope to selected geographical areas. All benchmarks have weights derived from the residential population grid [4].

**Table 1:** Basic information about the geographical areas that constitute our benchmarks. Each benchmark is located in compressed file (see column File), which is delivered with this paper.

**Benchmark**	**Number of DPs**	**Area [km**^**2**^**]**	**Population**	**File**
Slovakia	663,203	49,035	5,418,561	sr.zip
Žilina	79,612	6809	690,420	za.zip
Košice	9562	240	235,251	ke.zip
Partizánske	4873	301	47,801	pa.zip

### 1.1 Files organisation

Data set contains zipped files that constitute the basic benchmark Slovakia and three smaller benchmarks Žilina, Košice and Partizánske. Each benchmark is located in a separate zip archive and is published on-line together with this article.

All benchmarks are available in csv text editable files and in shapefiles that can be open inside a GIS tool. Below, we describe content and organisation of csv files. Shapefiles contain the same information.

After unzipping a file four folders are created:

• csv,
• gis,
• best_found_pmedian_solution,
• best_found_lexminimax_solution.

#### 1.1.1 Folder csv

In the folder ‘csv’ two csv text files are located, nodes.csv and edges.csv. These files contain all necessary information needed to construct a graph, representing the road network.

*Columns of file nodes.csv:*

***id*** - unique ID of nodes,

***latitude** -* latitude of nodes defined in WGS84 geographical coordination system,

***longitude** -* longitude of nodes defined in WGS84 geographical coordination system,

***type*** - type of the node (“dp” = demand point, “road” = node constituting the road network, “co” = node created to connect demand point to the road network),

***residential_populations*** – residential population associated with a node (weight of the demand point).

*Columns of file edges.csv:*

***origin** –* unique ID of the origin edge node (reference to *nodes.csv*),

***destination** –* unique ID of the destination edge node (reference to *nodes.csv*),

***length_meters** –* length of the edge in meters,

***maxspeed** –* speed limit [km/h] (“-1” if the value is unknown),

***oneway** - “yes” = unidirectional edge, “None” = bidirectional edge,*

***type** - type of the road according to the OSM (primary, residential, tertiary, motorway, trunk, unclassified, road, primary_link, secondary_link, motorway_link).*

#### 1.1.2 Folder GIS

Folder contains two files:

***nodes.shp*** – vector layer with all nodes,

***edges.shp*** – vector layer with all edges.

#### 1.1.3 Folder best_found_pmedian_solution

For benchmarks Žilina and Slovakia, that are too large to compute the optimal solution, we store the best found solutions for the p-median problem obtained by the adaptive aggregation framework proposed in [1]. For benchmarks Košice and Partizánske, the exact solutions are stored. Individual files contain the list of node IDs where the facilities are located. The p-value is used to distinguish which solutions are for which number of located facilities. Collection of shapefiles that can be used to visualize solutions in a GIS tool is located in the subfolder “*solution_shp*”. The file *objective_function_value.csv* contains the objective function values.

#### 1.1.4 Folder best_found_lexminimax_solution

For benchmark Žilina, we store here the best found solutions for the lexminimax problem obtained by the adaptive aggregation framework proposed in [1]. For benchmarks Košice and Partizánske, the exact solutions are stored. The organization of results is the same as in the p-median folder. The file *objective_function_value.csv* contains the maximal distance between a demand point and the closest facility and the value of the gini coefficient computed from distances between all demand points and closest facilities.

All benchmarks Slovakia, Žilina, Košice and Partizánske are stored in folders and files of identical structure. Benchmarks Slovakia and Žilina are too large to store the matrix of shortest path distances between all pairs of demand points in the computer memory, therefore, we publish only the graph structure. For benchmarks Košice and Partizánske we store in the file *Dmatrix.txt* the matrix computed on the entire graph Slovakia that has been used in computational experiments [1]. The matrix is stored in the distance value per line format. First two records define the dimensionality of the distance matrix. The distances are rounded to 100 m.

Benchmarks Košice and Partizánske can be derived from the benchmark Slovakia by spatially restricting the benchmark to a given subsets of nodes. To keep this link, the file *csv/nodes_in_slovakia.csv* is mapping the IDs of demand points to the IDs they have in the benchmark Slovakia. For this reason, the files with edges for Košice and Partizánske benchmarks are identical to the benchmark Slovakia and we did not store them. More benchmarks can be generated from the benchmark Slovakia, using attached python script *generate_Dmatrix.py*. Script expects as an input the file containing the list of IDs that should be used to form the benchmark. The output of the script consists of two files: *nodes.csv* and *Dmatrix.txt* (same format like for benchmarks Košice and Partizánske).

## 2 Experimental design, materials and methods

### 2.1 Materials

Delivered data set is a result of combining and processing the following data sets:

• OpenStretMap (OSM) [3],
• Residential population grid produced in [4].

### 2.2 Methods

In this section the process of generating benchmarks is described in detail.

#### 2.2.1 Data preparation

**Fig. 2:**
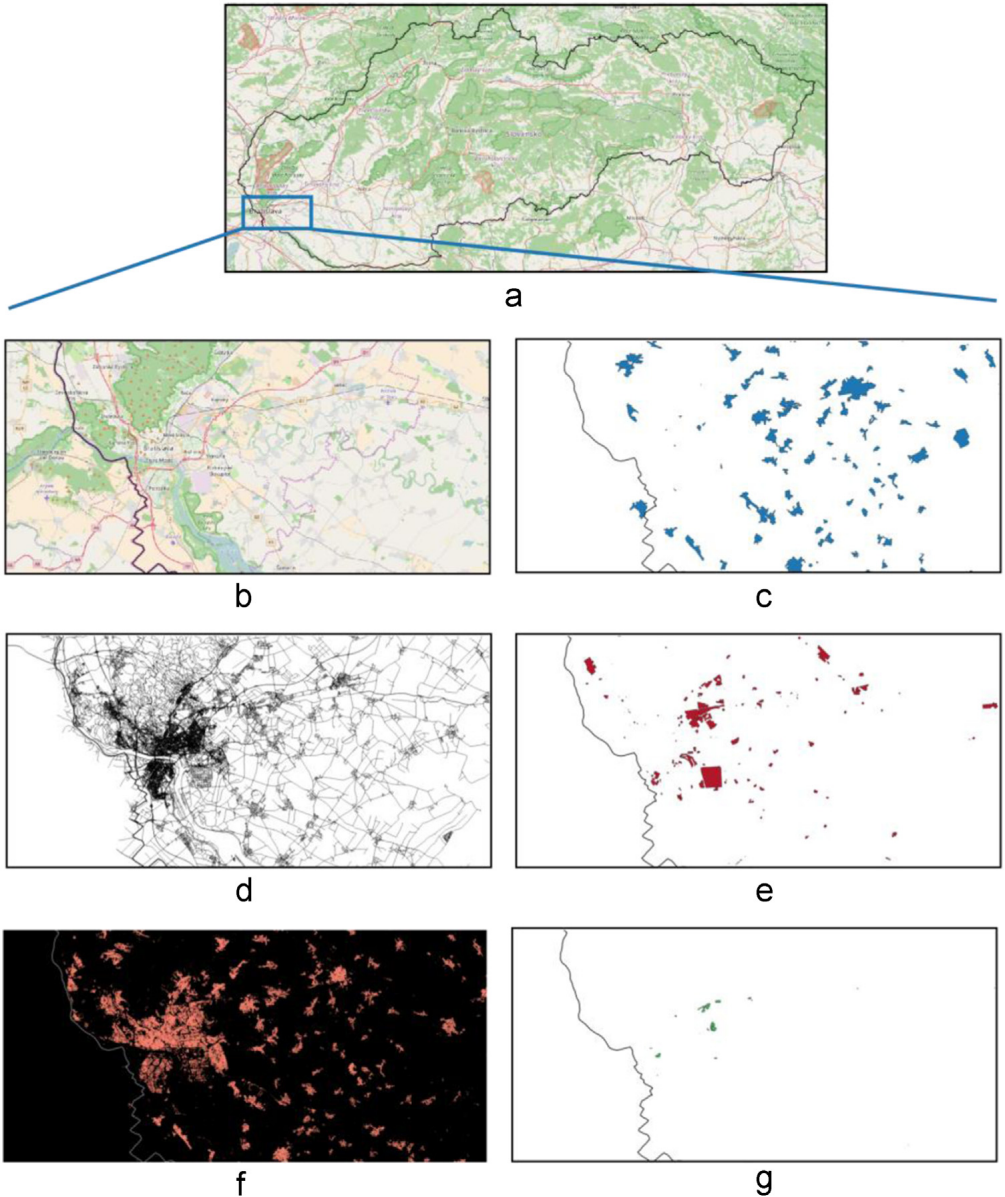
Illustration of the input OpenStreetMap data. (a) OpenStreetMap map of Slovakia with all layers. Zoom to the capital city of Bratislava: (b) all OSM layers, (c) residential areas, (d) road network, (e) industrial areas, (f) buildings and (g) commercial areas.

To capture the position of inhabitants independently on the time of the day, we extracted five basic OSM layers that allow estimating the positions of inhabitants when they are at home, at work and when they are travelling. Thus, to model demand points, we consider data layers describing positions of buildings, roads, residential, industrial and commercial areas (see Fig. 2). The graph used for calculating the travel distances is derived from the layer of public roads.

Demand points are generated in two steps: In the first step, 100×100 m spatial grid consisting of uniform square cells and covering the entire area of Slovakia is generated. In the second step, a demand point is situated as a centroid of each cell with a non-empty intersection with an OSM data layers (positions of buildings, roads, residential, industrial and commercial areas).

#### 2.2.2 Association of demand points with weights

**Fig. 3:**
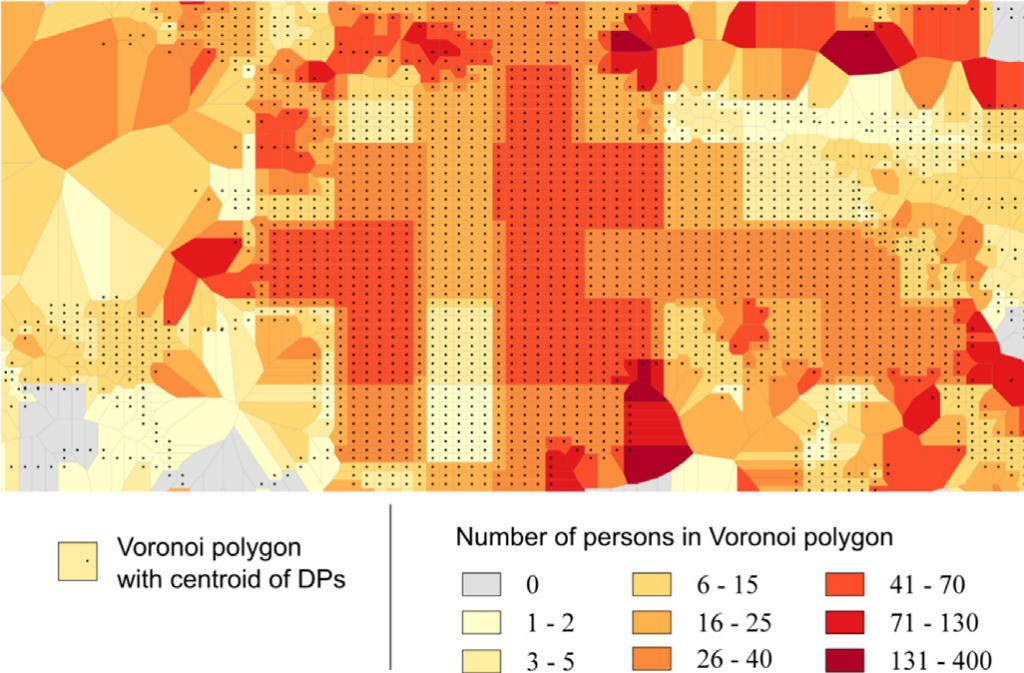
Illustration of the association of demand points with weights.

First, using demand points the Voronoi diagrams are created, where Voronoi polygons represent geographical areas associated with demand points. Second, we assign weights to demand points by intersecting Voronoi polygons with the population grid. The population (i.e. weight) assigned to a demand point is proportional to the population and to the area of the population grid cells intersecting the Voronoi polygon. Fig. 3 shows illustration of the association of demand points with weights.

#### 2.2.3 Graph model of the road network interconnecting all demand points

**Fig. 4:**
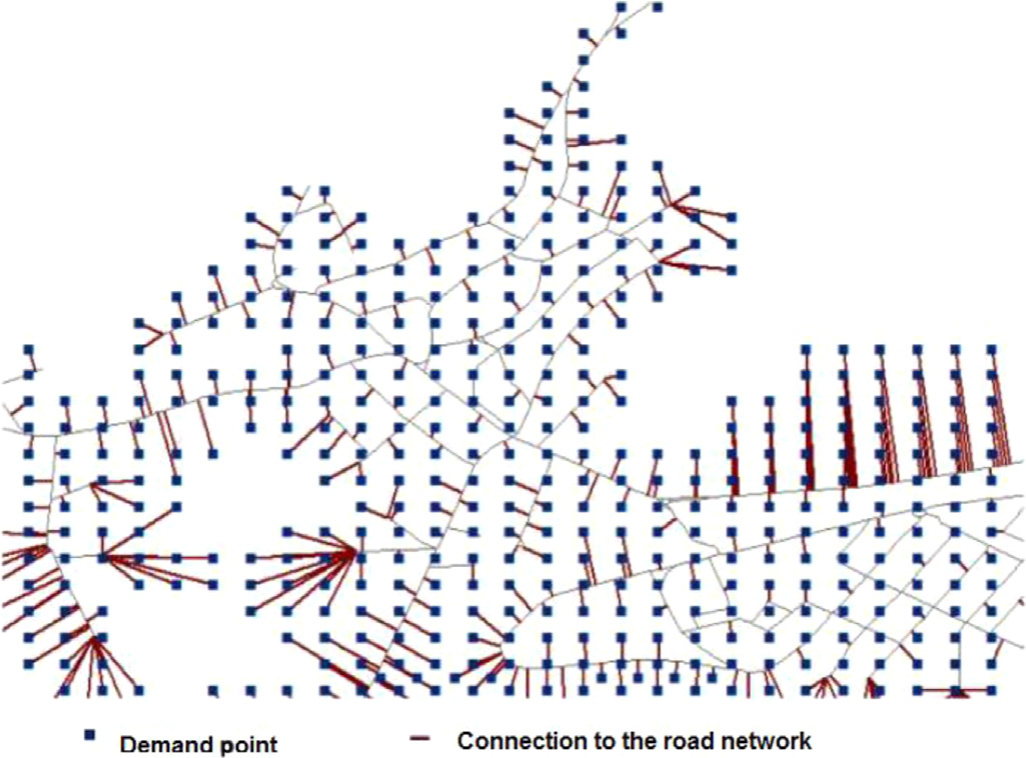
Illustration of interconnections between demand points and the road network.

Finally, we take the graph built in the data preparation phase and now all demand points are connected to the closest road segment. When it is necessary, the road segment is split by adding an intermediate node to minimise the length of the connection between the demand point and the road network (see Fig. 4). For the area of the Slovak Republic, by doing so, we obtained 1,956,067 nodes (including 663,203 demand points) and 2,080,694 links that all together constitute the graph.

#### 2.2.4 Quality of the OSM road network

As our data set is based on the OpenStreetMap data, therefore, the question of the quality of the OpenStreetMap road network of Slovakia should not be neglected. In Ref. [2], we conducted a study where we investigated this question (please refer to the Supplementary information file). To the best of our knowledge, this is the only one existing study that evaluates a quality of OSM road network for the area of the Slovak Republic.

**Fig. 5:**
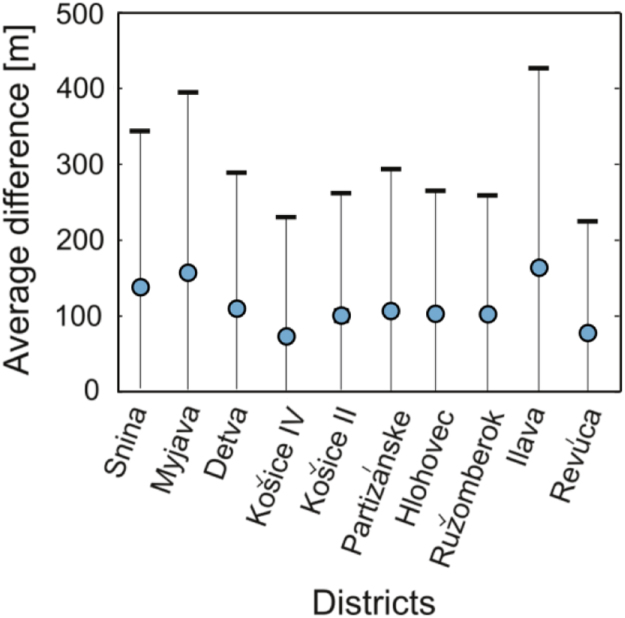
Average of absolute differences between lengths of the shortest paths calculated from OSM road network and those obtained from HERE Maps. Average is taken over 1000 node pairs in each district. Error bars are reflecting the standard deviation around the average. Figure is reproduced from the supplementary information file associated with the paper [2]. The average absolute difference is for all selected districts less than 180 m and in the majority of districts it is within 110 m. For more comprehensive analysis of the results see the supplementary information file of the paper [2].

The study compares OpenStreetMap with HERE Maps (previously known as OVI Maps or Nokia Maps, www.here.com). HERE Maps enables to obtain the shortest paths and travel times between pairs of road network vertices. Using the same graph model, which is delivered with this data paper, ten administrative districts of Slovakia are selected and for each of them 1000 different pairs of the road network vertices are randomly selected. Origin and destination vertices were always chosen from two different municipalities. Average values of the absolute differences between lengths of both (OSM and HERE Maps) shortest paths are displayed in Fig. 5.
